# Resolution of viral R-loop by DHX9 promotes hepatitis B virus replication

**DOI:** 10.1128/mbio.01449-26

**Published:** 2026-09-18

**Authors:** Yubo Pi, Huimin Luo, Kai Wang, Ni Tang, Ailong Huang, Yanmeng Chen

**Affiliations:** 1 Key Laboratory of Molecular Biology for Infectious Diseases, Ministry of Education, Department of Infectious Diseases, Institute for Viral Hepatitis, The Second Affiliated Hospital of Chongqing Medical University, Chongqing, China; 2 Youyang Hospital, a Branch of The First Affiliated Hospital of Chongqing Medical University, Chongqing, China; 3 Key Laboratory of Laboratory Medical Diagnostics, Ministry of Education, Department of Laboratory Medicine, Chongqing Medical University, Chongqing, China; Okayama University, Kurashiki, Okayama, Japan

**Keywords:** hepatitis B virus, DExH-box helicase 9, R-loop, transcription termination

## Abstract

Hepatitis B virus (HBV) is a hepatotropic DNA virus with a compact 3.2 kb genome that replicates via reverse transcription. HBV covalently closed circular DNA (cccDNA) transcription shares molecular features with R-loop. DExH-box helicase 9 (DHX9) has been shown to be associated with R-loop accumulation, genome instability, and HBV replication. However, the role of DHX9-mediated R-loop homeostasis in HBV transcription has not yet been elucidated. In this study, we identified a critical role of DHX9 in resolving R-loops at transcription termination during HBV transcription. We demonstrate that HBV replication promotes viral R-loop accumulation. DHX9 promotes HBV R-loop resolution, HBV cccDNA transcription termination, and subsequent nuclear export of the 3.5 kb HBV RNA, facilitating HBV DNA replication. These findings reveal DHX9-mediated R-loop resolution as a potential therapeutic target for antiviral intervention.

**IMPORTANCE:**

The persistence of HBV cccDNA poses a major challenge to achieving a cure for chronic hepatitis B. While R-loop structures have been implicated in the life cycles of viruses, their formation and functional significance in HBV replication have remained largely unexplored. This study provides the first evidence that the cellular helicase DHX9 resolves transcription-replication conflicts by dissolving R-loops formed during HBV replication. Our findings also identify DHX9-mediated R-loop resolution as a critical mechanism supporting the HBV life cycle. Consequently, targeting DHX9 presents a promising therapeutic strategy to silence the persistent HBV cccDNA reservoir.

## INTRODUCTION

Hepatitis B virus (HBV) is a hepatotropic DNA virus with a compact 3.2 kb genome that replicates via reverse transcription (1). The persistence of HBV covalently closed circular DNA (cccDNA) in the nucleus of HBV-infected hepatocytes represents the critical barrier to achieving viral elimination (2). Current antiviral therapies, including nucleos(t)ide analogs and interferon, fail to eliminate HBV cccDNA (3). Transcription of HBV cccDNA sustains HBV replication and facilitates viral rebound upon cessation of antiviral therapy (4). Consequently, there is an urgent need to develop novel therapeutic agents that silence HBV cccDNA transcription (5, 6).

R-loops are trimeric structures consisting of an RNA-DNA hybrid and a displaced DNA strand that are formed at promoters and sites of termination during transcription (7). R-loops participate in physiological regulation of gene expression, DNA damage repair, IgG switch recombination, and other functions. However, aberrant R-loop accumulation can lead to DNA damage, cellular stress responses, and genomic instability (8–10). Accumulating evidence indicates that R-loops are involved in the life cycles of multiple viruses. For example, human immunodeficiency virus type 1 (HIV-1) integrase can bind to the accumulated R-loop structures, facilitating viral integration into the host chromosome (11). Similarly, in human cytomegalovirus, a viral long non-coding RNA (lncRNA 4.9) forms an R-loop at the origin of lytic replication to initiate viral DNA synthesis (12). HBV replication involves a reverse transcription step that shares molecular features with R-loop-prone processes, and whether R-loops are formed during HBV cccDNA transcription remains unexplored.

R-loop homeostasis is maintained by R-loop-specific nucleases, such as RNase H1, RNase H2, and R-loop-specific helicases, such as DExH-box helicase 9 (DHX9) and senataxin (13, 14). DHX9 is a nuclear DExH-box RNA helicase that binds DNA:RNA hybrids to resolve R-loop structures during DNA replication and transcription (15, 16). DHX9 deficiency is associated with R-loop accumulation, RNA polymerase II stabilization, and read-through transcripts, which have shed light on the importance of DHX9 in R-loop-mediated genomic instability (15, 17). DHX9 helicase also promotes R-loop suppression and transcriptional termination (13). Furthermore, DHX9 modulates the replication of multiple viruses, including Zika virus (ZIKV) and HIV (18, 19). Our previous studies indicate that HBx-mediated DHX9 upregulation contributes to HBV replication by attenuating the anti-HBV efficacy of apolipoprotein B mRNA editing enzyme catalytic subunit 3B (20, 21). However, the role of DHX9-mediated R-loop homeostasis in HBV transcription has not yet been elucidated.

In this study, we found a critical role of DHX9 in resolving R-loops at transcription termination during HBV transcription. We demonstrate that HBV replication induces viral R-loop formation, and that DHX9 promotes HBV R-loop resolution, facilitating HBV cccDNA transcription termination and subsequent nuclear export of the 3.5 kb HBV RNA. These findings identify DHX9-mediated R-loop resolution as a potential therapeutic target for antiviral intervention.

## RESULTS

### Overexpression of DHX9 promotes HBV replication

**Fig 1:**
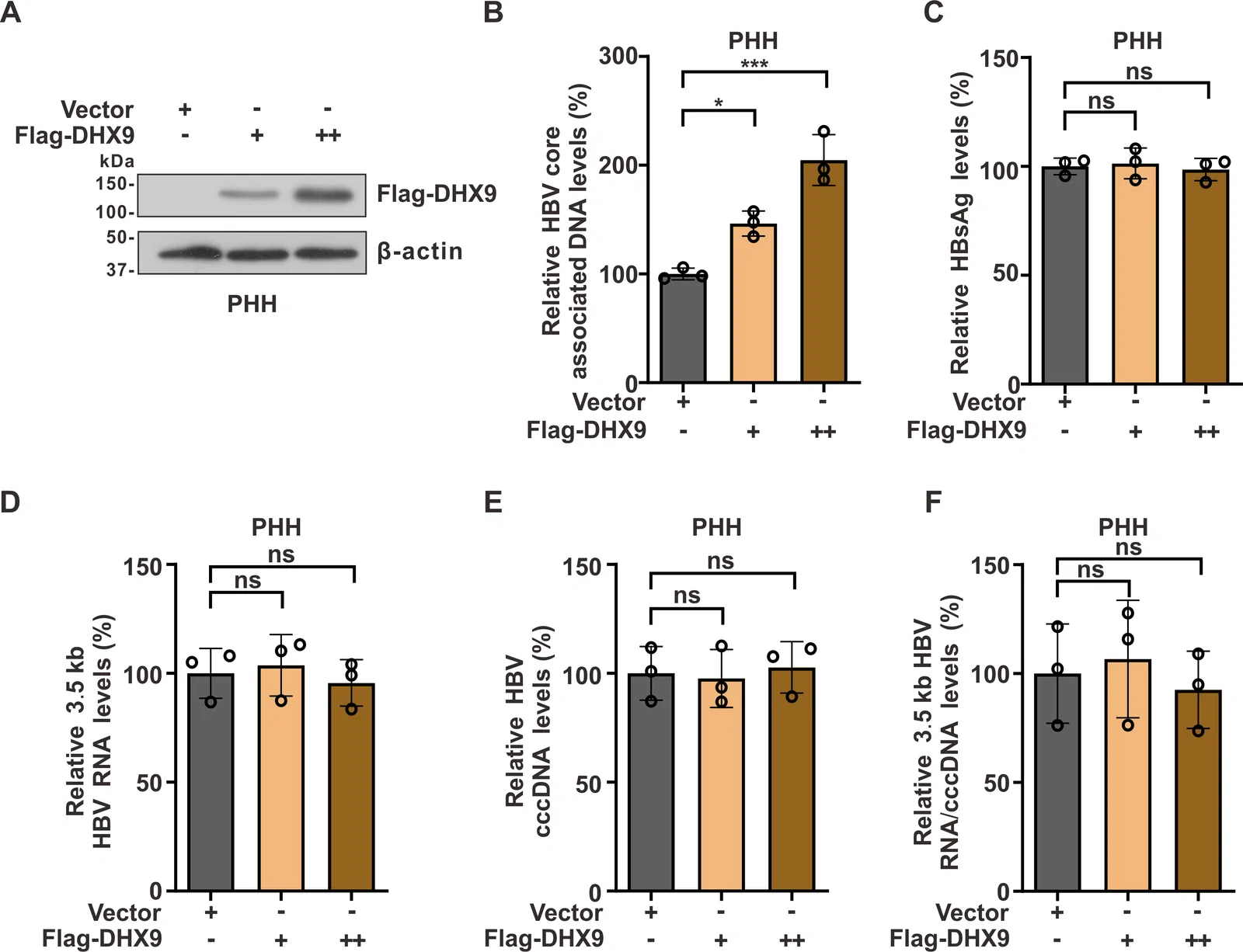
Overexpression of DHX9 promotes HBV replication. PHHs were infected with HBV particles. After 24 h post-infection, the cells were transfected with increasing amounts of Flag-tagged DHX9 plasmids. (**A**) Western blot analysis of DHX9 expression. (**B**) Quantification of HBV core-associated DNA levels using qPCR. (**C**) The levels of HBsAg in culture medium were determined by ELISA. (**D**) Quantification of 3.5 kb HBV RNA levels using RT-qPCR. (**E**) Quantification of HBV cccDNA levels using qPCR. (**F**) The ratio of 3.5 kb HBV RNA to HBV cccDNA was calculated to assess HBV cccDNA transcriptional activity. Data represent mean ± SD from three biological replicates. *P < 0.05, ***P < 0.001, ns: no significance, one-way ANOVA with Tukey’s *post hoc* test.

We first investigated the potential effect of DHX9 on HBV replication in HBV-infected HepG2-NTCP cells. The cells were transfected with increasing amounts of DHX9 expression plasmids (Fig. S1A). Overexpression of DHX9 significantly enhanced the levels of HBV core-associated DNA (Fig. S1B). In contrast, DHX9 overexpression did not alter the levels of HBsAg (Fig. S1C), 3.5 kb HBV RNA (Fig. S1D), HBV cccDNA (Fig. S1E), and the ratio of 3.5 kb HBV RNA to HBV cccDNA (Fig. S1F). To validate this finding, we extended our analysis to primary human hepatocytes (PHHs) with HBV infection. Consistent with the observations in HepG2-NTCP cells, DHX9 overexpression in PHHs (Fig. 1A) promoted HBV core-associated DNA replication (Fig. 1B) without altering the levels of HBsAg (Fig. 1C), 3.5 kb HBV RNA (Fig. 1D), HBV cccDNA (Fig. 1E), and the ratio of 3.5 kb HBV RNA to HBV cccDNA (Fig. 1F). Collectively, these results demonstrate that DHX9 facilitates HBV DNA replication independent of HBV cccDNA transcription.

### Knockdown of DHX9 restricts HBV replication

**Fig 2:**
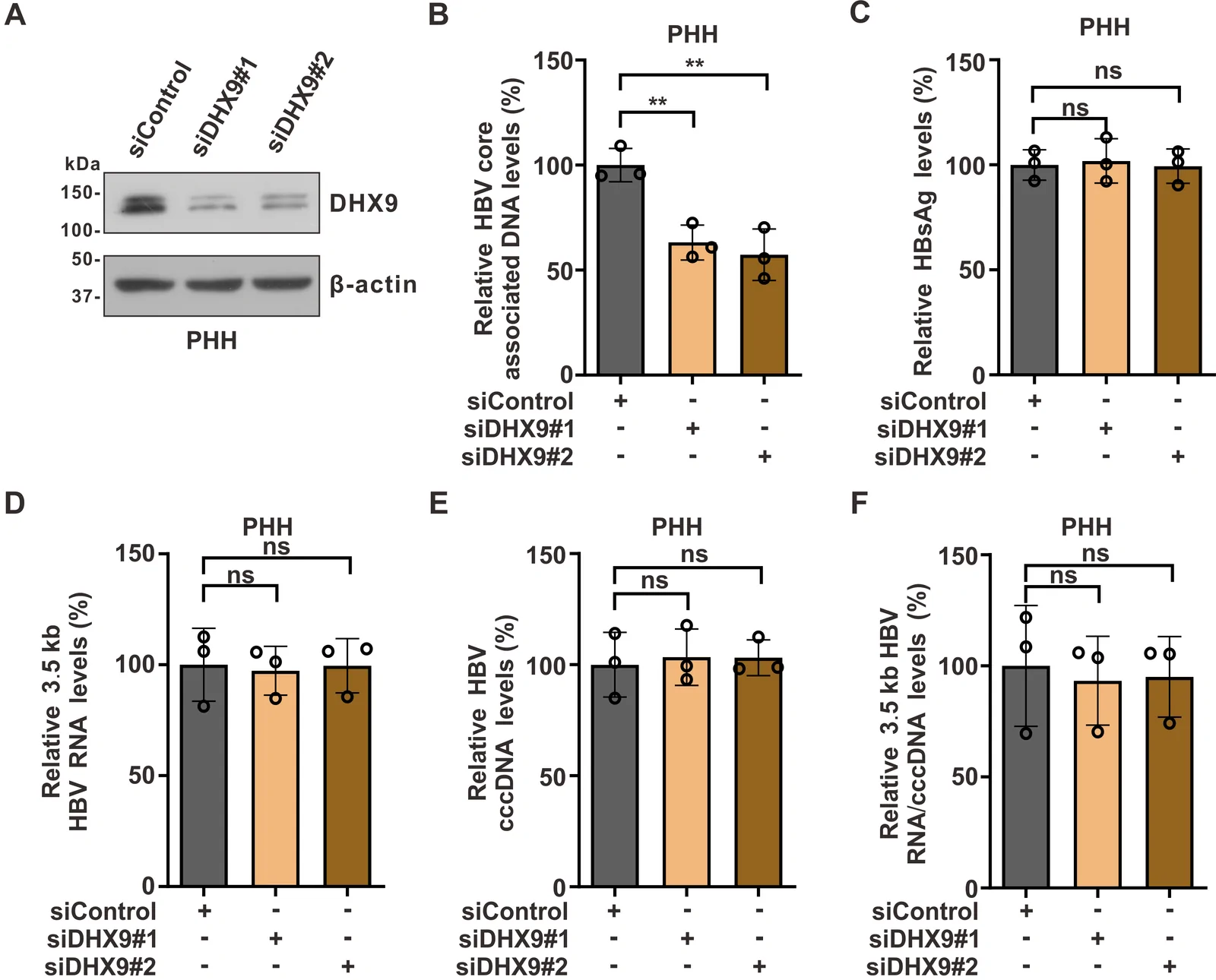
Knockdown of DHX9 inhibits HBV replication. PHHs were infected with HBV particles. After 24 h post-infection, the cells were transfected with siDHX9 or siControl as indicated. (**A**) Western blot analysis of DHX9 expression. (**B**) Quantification of HBV core-associated DNA levels using qPCR. (**C**) The levels of HBsAg in culture medium were determined by ELISA. (**D**) Quantification of 3.5 kb HBV RNA levels using RT-qPCR. (**E**) Quantification of HBV cccDNA levels using qPCR. (**F**) The ratio of 3.5 kb HBV RNA to HBV cccDNA was calculated to assess HBV cccDNA transcriptional activity. Data represent mean ± SD from three biological replicates. **P < 0.01, ns: no significance, one-way ANOVA with Tukey’s *post hoc* test.

Next, to further validate the role of DHX9 in HBV replication, endogenous DHX9 expression was knocked down in HepG2-NTCP cells using siDHX9 (Fig. S2A). Silencing of DHX9 significantly reduced HBV core-associated DNA levels (Fig. S2B) but did not alter the levels of HBsAg (Fig. S2C), 3.5 kb HBV RNA (Fig. S2D), HBV cccDNA (Fig. S2E), and the ratio of 3.5 kb HBV RNA to HBV cccDNA (Fig. S2F). The results were further confirmed in HBV-infected PHHs (Fig. 2A through F). These results demonstrate that DHX9 promotes HBV DNA replication at the post-transcriptional stage of HBV cccDNA.

### HBV replication induces R-loop formation

**Fig 3:**
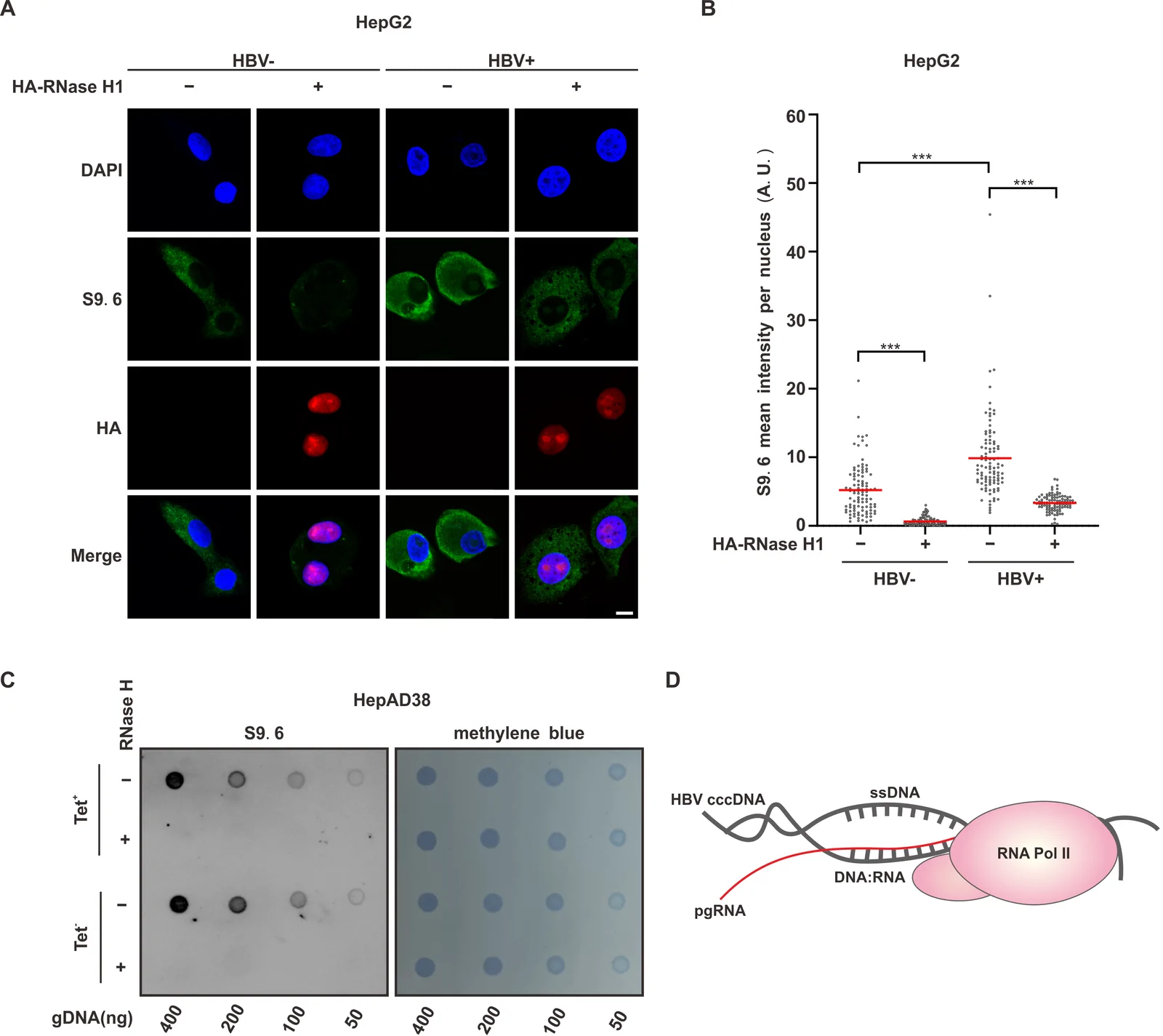
HBV replication induced R-loop accumulation. (**A**) R-loops in HBV-replicated HepG2 cells were detected by immunofluorescence. (**B**) Quantification of R-loops detected by immunofluorescence in HBV-replicated HepG2 cells. (**C**) The R-loops in HepAD38 cells were detected by Dot blot. (**D**) Schematic model of R-loop structure formed during HBV transcription. Scale bar, 10 µm. DAPI, 4′,6-diamidino-2-phenylindole. ***P < 0.001, one-way ANOVA with Tukey’s *post hoc* test.

Given that the transcription and nuclear export of HBV pgRNA are vital to HBV DNA replication (22), we hypothesized that HBV R-loops could form between the nascent single-stranded HBV pgRNA and the double-stranded HBV cccDNA template during viral transcription. We transfected HepG2 cells with the HBV 1.1-mer plasmid and assessed R-loop levels by immunofluorescence using the S9.6 antibody. The results showed a marked increase in R-loop signals upon HBV replication (Fig. 3A and B). Furthermore, overexpression of RNase H1 in HBV-infected HepG2 cells effectively reduced the elevated R-loop levels, confirming their identity as DNA:RNA hybrids (Fig. 3A and B). Dot blot analysis further revealed that HBV replication led to increased R-loop formation, and HBV-induced R-loops were sensitive to RNase H treatment (Fig. 3C). Collectively, these findings demonstrate that HBV transcription promotes R-loop formation.

Next, to determine whether the HBV-associated R-loops originate from HBV transcription, we performed DNA:RNA immunoprecipitation followed by sequencing (DRIP-seq) (Fig. S3A). The results revealed significant R-loop signals across the HBV genome (Fig. S3B). Furthermore, these signals were markedly reduced upon RNase H1 expression (Fig. S3B). These results indicated that viral R-loops are formed during HBV transcription (Fig. 3D).

### DHX9 depletion induces R-loop accumulation during HBV replication

**Fig 4:**
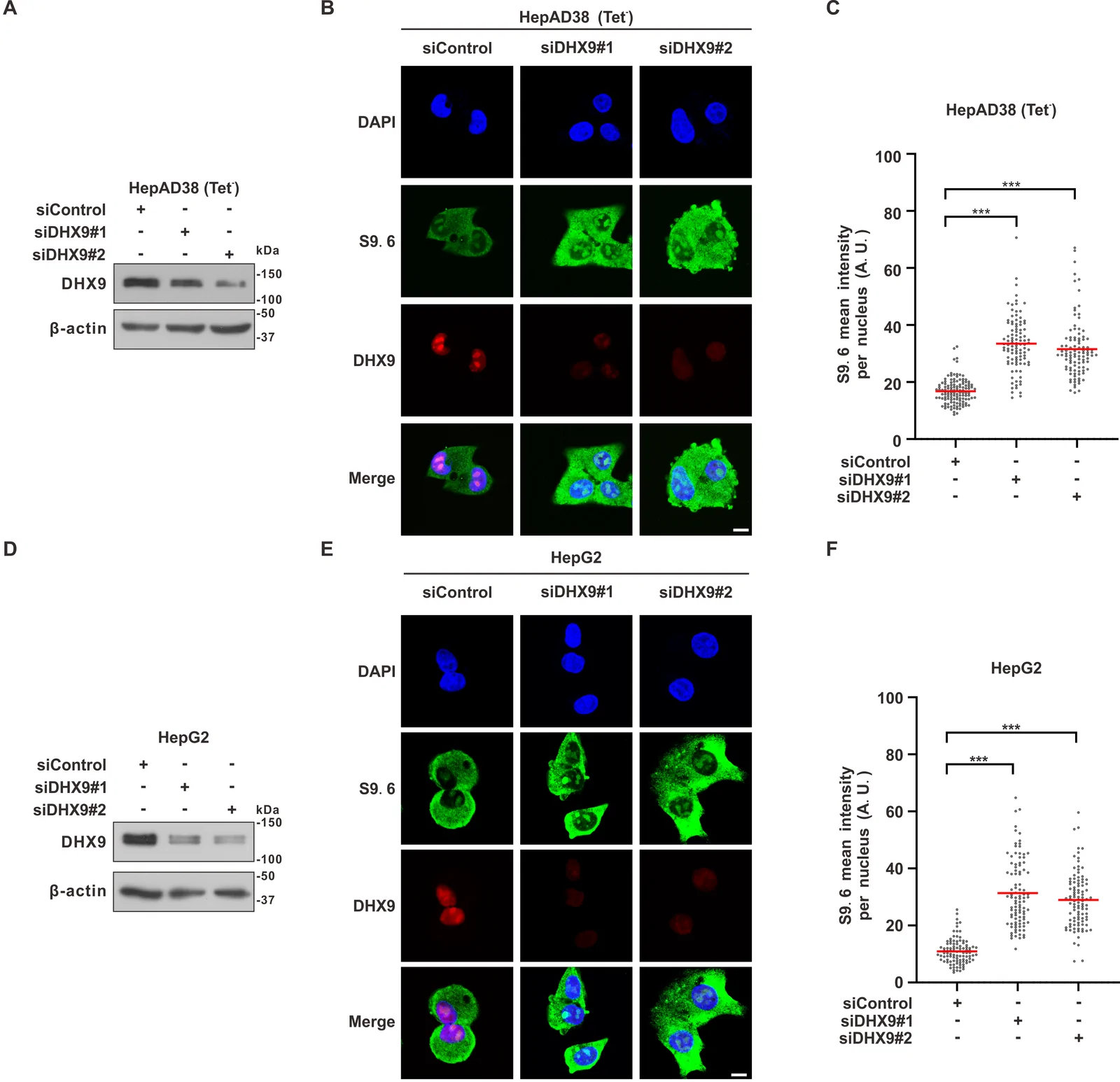
Knockdown of DHX9-induced R-loop accumulation. (**A–C**) HepAD38 cells in tetracycline-free medium were transfected with siDHX9 or siControl as indicated. (**A**) Western blot analysis of DHX9 expression. (**B**) R-loops in HepAD38 cells were detected by immunofluorescence. (**C**) Quantification of R-loops detected by immunofluorescence in HepAD38 cells. (**D–F**) HepG2 cells were transfected with HBV plasmid, siDHX9, or siControl as indicated. (**D**) Western blot analysis of DHX9 expression. (**E**) R-loops were detected by immunofluorescence in HepG2 cells. (**F**) Quantification of R-loops detected by immunofluorescence in HepG2 cells. Scale bar, 10 µm. DAPI, 4′,6-diamidino-2-phenylindole. ***P < 0.001, one-way ANOVA with Tukey’s *post hoc* test.

**Fig 5:**
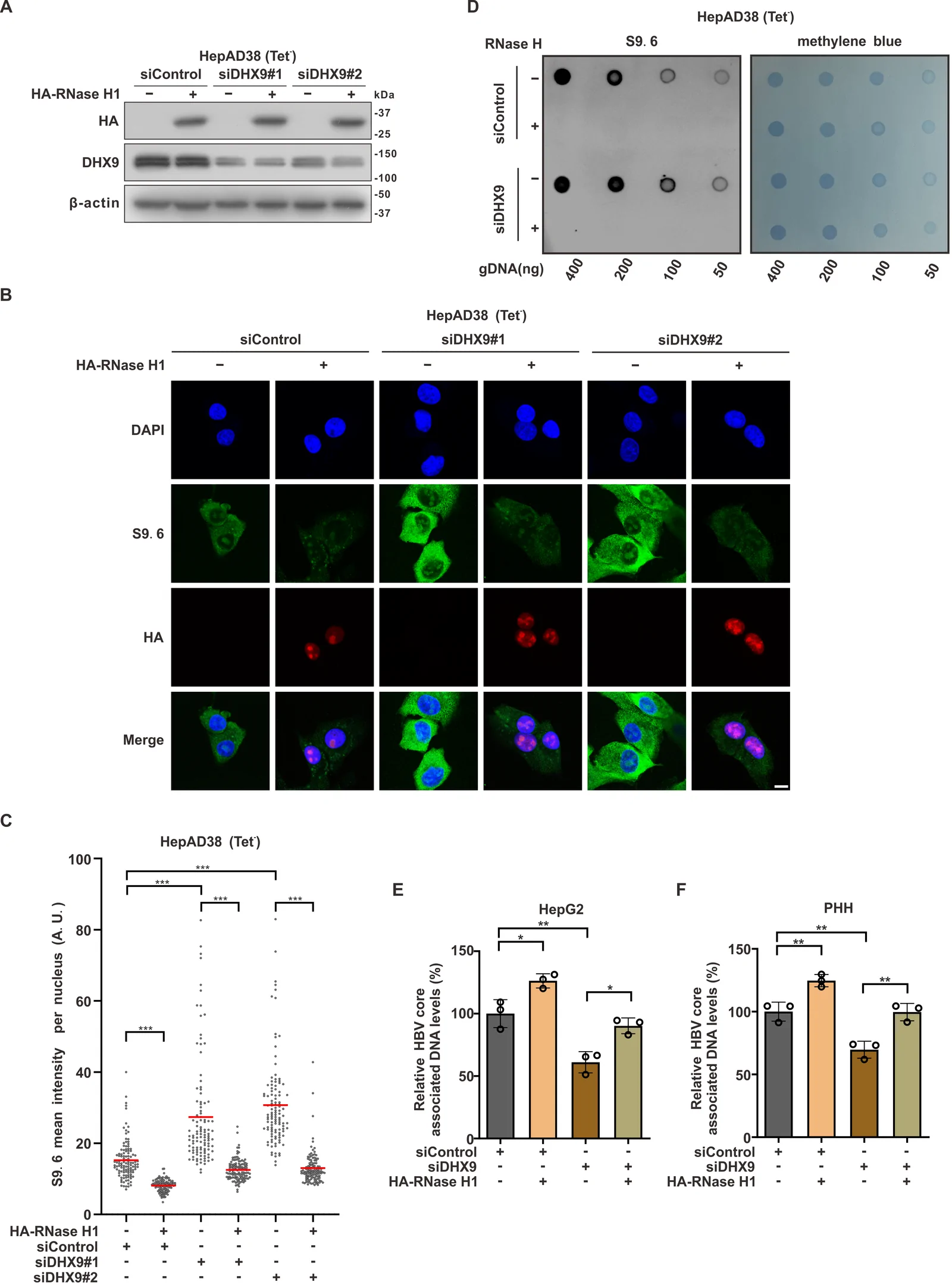
RNase H treatment reduces R-loop accumulation and promotes HBV replication. (**A**) Western blot analysis of protein expression in HepAD38 cells co-transfected with siDHX9 and an HA-RNase H1 expression plasmid. (**B**) R-loops in HepAD38 cells co-transfected with siDHX9 and an HA-RNase H1 expression plasmid were detected by immunofluorescence. (**C**) Quantification of R-loops detected by immunofluorescence in panel **B**. (**D**) Dot blot analysis of R-loop levels in HepAD38 cells using the S9.6 antibody. (**E**) HepG2 cells were transfected with HBV 1.1-mer plasmid, HA-tagged RNase H1 plasmid, and siRNA targeting DHX9 (siDHX9) or control siRNA (siControl). Quantification of HBV core-associated DNA levels using qPCR. (**F**) PHHs were infected with HBV particles. At 24 h post-infection, the cells were transfected with HA-tagged RNase H1 plasmid and siRNA targeting DHX9 (siDHX9) or control siRNA (siControl). Quantification of HBV core-associated DNA levels using qPCR. Data represent mean ± SD from three biological replicates. Scale bar, 10 µm. DAPI, 4′,6-diamidino-2-phenylindole. *P < 0.05, **P < 0.01, ***P < 0.001, one-way ANOVA with Tukey’s *post hoc* test.

Based on the observation that HBV replication induces R-loop formation, we hypothesized that DHX9 regulates the generation of R-loops during HBV transcription. We knocked down endogenous DHX9 expression in HepAD38 (Tet^–^) cells using siDHX9 (Fig. 4A). Immunofluorescence with the S9.6 antibody revealed that DHX9 depletion led to a marked increase in R-loop levels in HBV-replicating HepAD38 cells (Fig. 4B and C). Similar results were also observed in the HBV-replicating HepG2 cells (Fig. 4D through F). Notably, this elevation was effectively reversed by RNase H1 expression (Fig. 5A through C), indicating that depletion of DHX9 triggers R-loop accumulation. Consistent with these findings, dot blot analysis also confirmed increased R-loop signals upon DHX9 silencing (Fig. 5D). We next examined whether DHX9-mediated R-loop accumulation regulates HBV replication. In both HepG2 cells and PHHs, RNase H1 overexpression reversed the decrease in HBV core-associated DNA induced by DHX9 silencing (Fig. 5E and F), suggesting that DHX9 promotes HBV replication through regulating R-loop homeostasis.

### DHX9 prevents viral R-loop accumulation at the transcription termination site during HBV transcription

**Fig 6:**
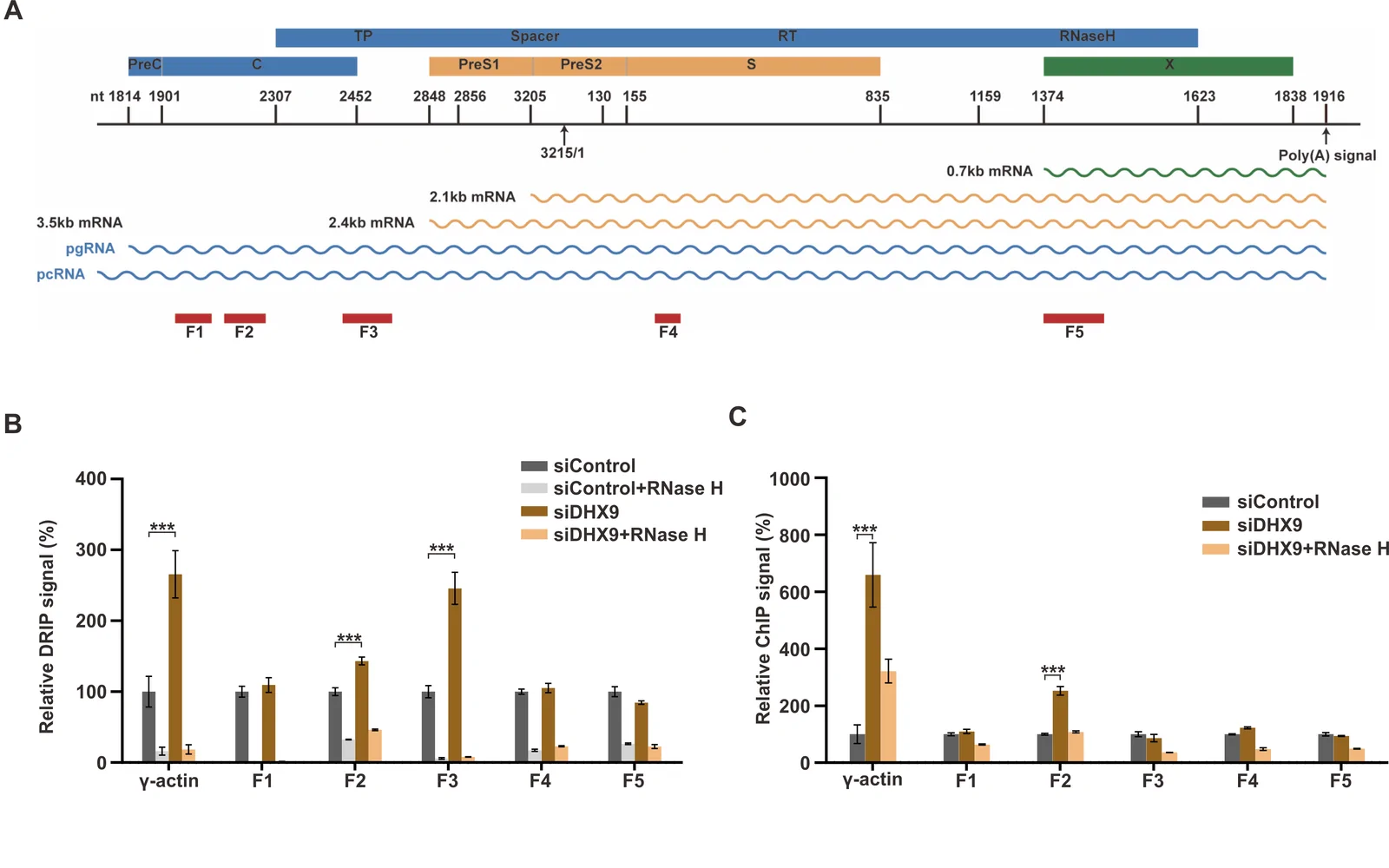
Depletion of DHX9 induces R-loop accumulation downstream of the HBV poly(A) site. (**A**) Schematic diagram illustrating the primer design spanning the HBV genome for DRIP-qPCR and ChIP-qPCR. (**B**) DRIP-qPCR analysis of R-loop levels at the HBV genome in DHX9-deficient HepAD38 cells. (**C**) ChIP-qPCR analysis of RNA polymerase II occupancy at the HBV genome in DHX9-deficient HepAD38 cells. Data represent mean ± SD from three biological replicates. ***P < 0.001, one-way ANOVA with Tukey’s *post hoc* test.

To detect the location of the R-loop on the HBV genome, we designed five pairs of primers covering different regions (Fig. 6A). DRIP-qPCR analysis demonstrated that DHX9 knockdown specifically enhanced viral R-loop accumulation downstream of the poly(A) signal sites (F2-F3), and this effect was abolished by RNase H treatment (Fig. 6B). Given that efficient transcriptional termination of the 3.5 kb HBV RNA depends on the recognition of the poly(A) signal by RNA polymerase II (Pol II), we performed chromatin immunoprecipitation-qPCR (ChIP-qPCR) to assess Pol II occupancy on the HBV genome. The results showed that DHX9 depletion led to significant retention of Pol II at the transcriptional termination region (Fig. 6C), suggesting that DHX9 facilitates transcription termination of HBV RNAs. Collectively, these data demonstrate that DHX9 prevents R-loop accumulation at the transcription termination during HBV transcription, alleviating R-loop-driven transcriptional stress and facilitating viral replication.

### DHX9 promotes nucleocytoplasmic export of 3.5 kb HBV RNA

**Fig 7:**
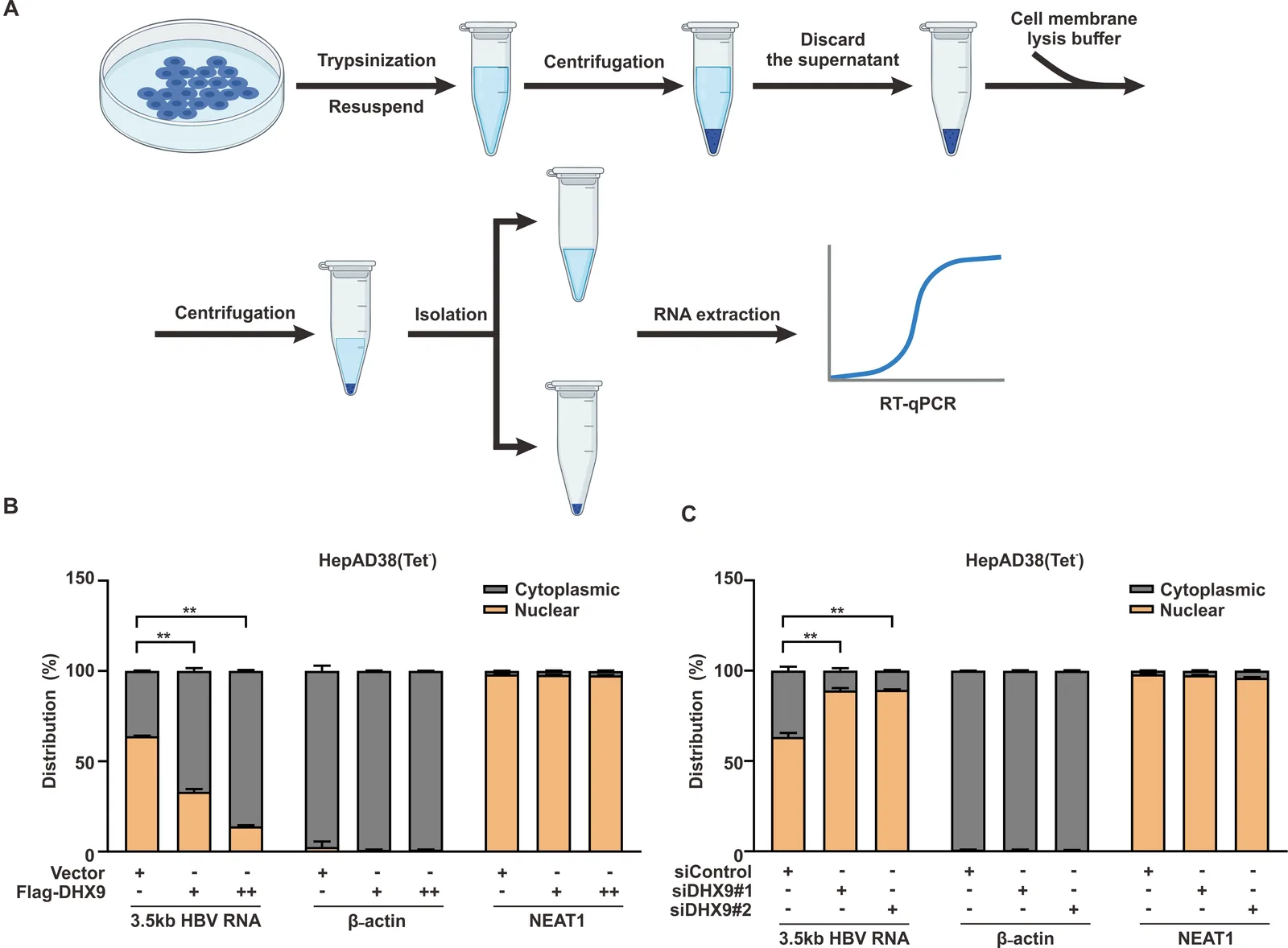
DHX9 promotes nucleocytoplasmic transport of 3.5 kb HBV RNA. (**A**) Schematic diagram of nuclear and cytoplasmic RNA extraction using subcellular fractionation. (**B**) Nucleoplasmic quantitation of 3.5 kb HBV RNA in DHX9-overexpressed HepAD38 cells. (**C**) Nucleoplasmic quantitation of 3.5 kb HBV RNA in DHX9-silenced HepAD38 cells. Data represent mean ± SD from three biological replicates. **P < 0.01, one-way ANOVA with Tukey’s *post hoc* test.

Considering that nucleocytoplasmic export of 3.5 kb HBV RNA is vital to the production of HBV core-associated DNA, we hypothesized that DHX9 may facilitate transcription termination and nucleocytoplasmic transport of 3.5 kb HBV RNA. To test this hypothesis, we examined the effect of DHX9 expression on the subcellular distribution of the 3.5 kb viral transcript (Fig. 7A). Subcellular fractionation analysis revealed that DHX9 overexpression significantly enhanced the accumulation of 3.5 kb HBV RNA in the cytoplasm (Fig. 7B). Conversely, DHX9 knockdown led to nuclear retention of the viral transcript (Fig. 7C). These findings indicate that DHX9 promotes the nuclear export of 3.5 kb HBV RNA, which is critical for the synthesis of viral DNA in HBV replication.

### DHX9 knockdown induces R-loop accumulation and inhibits HBV replication *in vivo*

**Fig 8:**
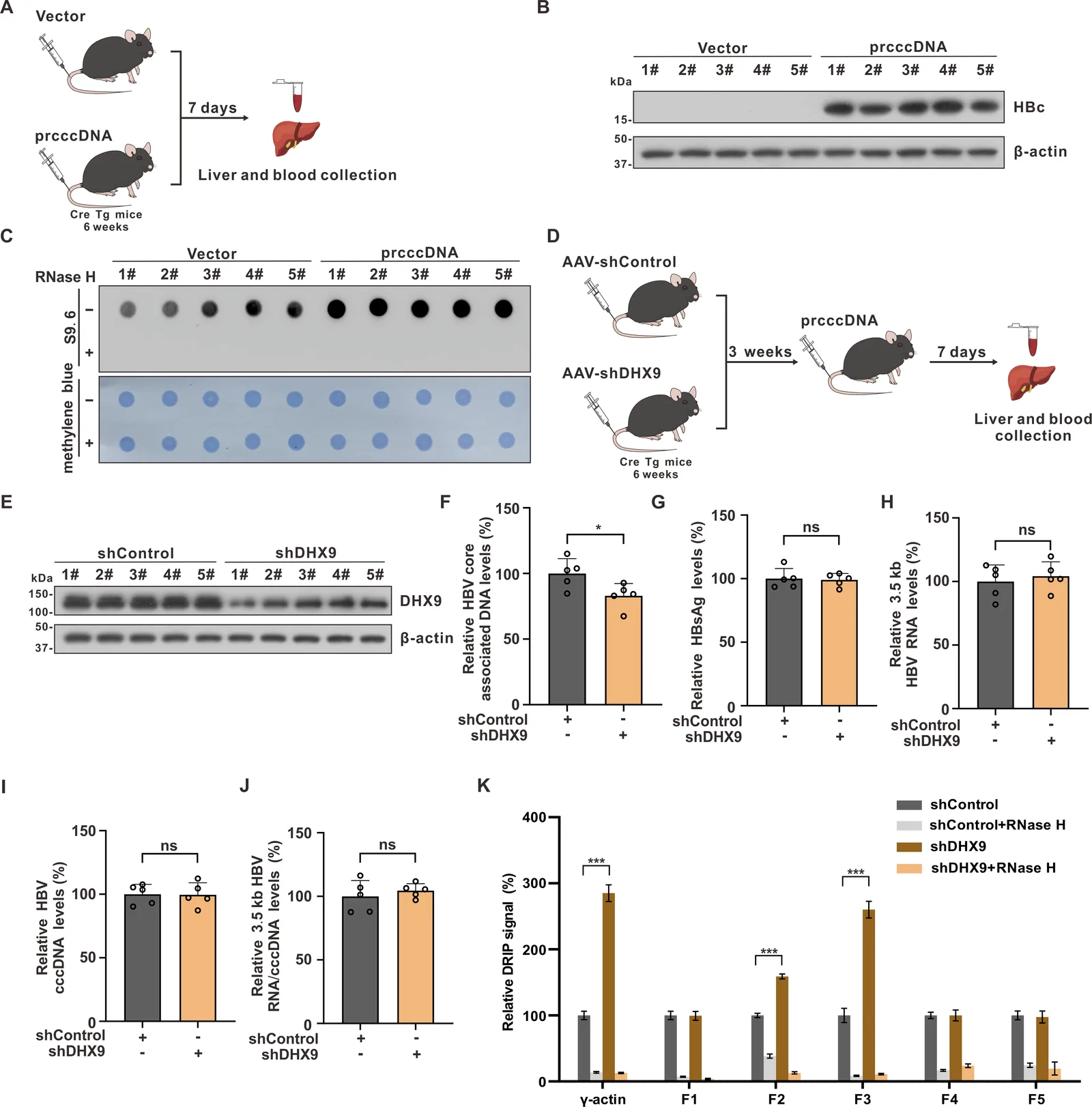
DHX9 knockdown induces R-loop accumulation and inhibits HBV replication in prcccDNA-delivered mice. (**A**) The schematic depiction of experiments in prcccDNA-delivered *Alb-Cre* Tg mice. The mice were randomly divided into two groups: the prcccDNA-delivered group and the control group. The prcccDNA plasmid or vector plasmid was injected into *Alb-Cre* Tg C57BL/6 mice via the tail vein using a hydrodynamic model. Liver and blood samples were collected at 7 days post-injection. (**B**) HBc protein levels were analyzed by Western blot to assess the effect of HBV infection. (**C**) Dot blot analysis of the R-loops in HBV-delivered mice. (**D**) The schematic depiction of experiments in DHX9-silenced *Alb-Cre* Tg mice. The *Alb-Cre* Tg mice were injected with AAV-shControl or AAV-shDHX9 through tail veins. At 3 weeks post-injection, the mice were injected with prcccDNA plasmid or vector plasmid through tail veins. Liver tissue and blood samples from mice were collected at 7 days post-injection. (**E**) Western blot analysis of DHX9 expression to confirm protein knockdown efficiency. (**F**) Quantification of HBV core-associated DNA levels in mouse liver tissues using qPCR. (**G**) The levels of HBsAg in serum samples were determined by ELISA. (**H**) Quantification of 3.5 kb HBV RNA levels in mouse liver tissues. (**I**) Quantification of HBV cccDNA levels in mouse liver tissues using qPCR. (**J**) The ratio of 3.5 kb HBV RNA to HBV cccDNA was calculated to assess HBV cccDNA transcriptional activity. (**K**) DRIP-qPCR analysis of R-loop levels at the HBV genome in RNase H-treated liver tissues. Data represent mean ± SD from five biological replicates. *P < 0.05, ***P <0.001, ns: no significance, one-way ANOVA with Tukey’s *post hoc* test was used to analyze data in (**K**). Other data were analyzed using a two-tailed unpaired Student’s *t* test.

To assess R-loop dynamics *in vivo*, HBV-delivered *Alb/Cre* transgenic (Tg) mouse models were constructed via hydrodynamic injection of precursor recombinant cccDNA plasmids (prcccDNA) (Fig. 8A) (23). HBV infection was confirmed by Western blot (Fig. 8B). Dot blot analysis revealed that HBV replication induced R-loop accumulation in mouse livers, and accumulated R-loops were sensitive to RNase H treatment (Fig. 8C), indicating that HBV infection induces R-loop accumulation.

To evaluate the impact of DHX9 on HBV replication and R-loop formation, we established HBV-delivered mice with DHX9 knockdown (23). *Alb/Cre* Tg mice were injected with AAV-shDHX9 or AAV-shControl via tail vein. After 3 weeks, the mice were hydrodynamically injected with the prcccDNA plasmid. Liver tissues and serum samples were collected on day 7 post-plasmid injection (Fig. 8D). Effects of DHX9 knockdown in liver tissues were confirmed by Western blot (Fig. 8E). Knockdown of DHX9 expression significantly reduced the levels of HBV core-associated DNA (Fig. 8F) but did not affect serum HBsAg levels (Fig. 8G). Furthermore, DHX9 knockdown had no significant effect on the levels of intrahepatic 3.5 kb HBV RNA (Fig. 8H), HBV cccDNA (Fig. 8I), or the ratio of 3.5 kb HBV RNA to HBV cccDNA (Fig. 8J). Subsequent DRIP-qPCR analysis showed that DHX9 knockdown led to significant R-loop accumulation downstream of the viral poly(A) signal site (Fig. 8K), indicating that DHX9 facilitates HBV replication by resolving R-loops downstream of the poly(A) signal region in the transcription termination region of HBV transcription.

## DISCUSSION

R-loops, naturally occurring three-stranded nucleic acid structures comprising a DNA:RNA hybrid and a displaced single-stranded DNA, play important roles in regulating gene expression and maintaining genomic stability (12). While their physiological formation is involved in transcription regulation and DNA repair, aberrant accumulation of R-loops is widely recognized as a source of genomic instability, often associated with DNA damage and disrupted RNA splicing (24). Here, we identify the formation of R-loops as a novel feature of HBV transcription from HBV cccDNA. We demonstrate that the host RNA helicase DHX9 resolves these viral R-loops, specifically facilitating the dissociation of RNA polymerase II at the viral poly(A) signal to ensure proper transcription termination. Importantly, this DHX9-mediated resolution is a prerequisite for the subsequent nuclear export of the 3.5 kb HBV RNA, thereby promoting the synthesis of HBV core-associated DNA and overall replication. Our work delineates a previously unrecognized post-transcriptional checkpoint in the HBV life cycle, governed by DHX9-dependent R-loop metabolism.

The RNA helicase DHX9 exhibits a dual, context-dependent role during viral infections. It serves as a restriction factor for Epstein-Barr virus (EBV) by bolstering interferon-stimulated gene-mediated innate immunity against lytic infection, for myxoma virus (MYXV) by forming cytoplasmic antiviral granules that suppress late viral protein synthesis, and for ZIKV by partnering with interleukin enhancer-binding factor 3 to promote dicer endoribonuclease-dependent antiviral RNAi against viral stem-loop B RNAs (18, 25, 26). Conversely, for HIV-1, DHX9 binds the primer binding site segment of viral RNA to promote cap hypermethylation, thereby driving specialized translation of viral proteins and enhancing replication (27). This functional dichotomy likely reflects virus-specific strategies and distinct interactions with host or viral components (28, 29). In the context of HBV, earlier work from our group and others established that the viral HBx protein upregulates DHX9 expression and that DHX9 promotes HBV replication, primarily through non-enzymatic functions such as RNA binding or signaling modulation (21, 30). Here, we extend these findings by demonstrating that DHX9 operates at the transcriptional level in HBV-infected cells via its canonical helicase activity. Specifically, we show that this enzymatic function is required for resolving HBV-induced R-loops, thereby promoting viral replication. Compared to other viral systems where DHX9’s role is largely defined by its RNA-sensing or adaptor capabilities, our study reveals a distinct mechanism by which HBV co-opts the helicase core to resolve transcription-associated R-loops. These findings define DHX9 as a pro-viral host factor that acts at a critical post-transcriptional checkpoint, uncovering a previously uncharacterized layer of host-pathogen interaction in which HBV hijacks host nucleic acid metabolism for efficient replication.

Transcription of the HBV pgRNA by RNA polymerase II from the HBV cccDNA represents a critical step in the HBV life cycle (31). Termination of all HBV RNA transcripts is directed by a polyadenylation signal consisting of a UAUAAA motif and downstream UGU repeats (32, 33). Although *in vitro* studies have shown that accurate 3′-end processing of HBV RNAs depends on sequences flanking this signal, the molecular mechanisms governing transcription termination and the molecular mechanisms governing this process and its functional impact on HBV replication remain incompletely understood (34). Recent studies highlight the pivotal role of R-loops in regulating transcription termination in cellular genes (35). Aberrant accumulation of R-loops is closely linked to dysregulated gene expression, DNA damage, and disease pathogenesis (36). Notably, several viruses, including human papillomavirus (HPV), EBV, and HIV, manipulate R-loop formation on host or viral chromatin to facilitate their replication and pathogenesis (11, 37–39). However, whether HBV replication induces R-loop formation and how such structures might influence the viral life cycle currently remain elusive. Here, we demonstrate for the first time that HBV transcription itself generates R-loops and that resolution of these structures is required for efficient transcription termination. These findings unveil an R-loop-dependent layer of post-transcriptional regulation that is co-opted by HBV to sustain its replication, thereby revealing a novel mechanism of HBV dependence on host nucleic acid metabolism.

DHX9, an integral component of the RNA polymerase II holoenzyme, plays a crucial role in co-transcriptional pre-mRNA processing and termination (40). Its helicase activity enables it to resolve aberrant nucleic acid secondary structures, including DNA:RNA hybrids and G-quadruplexes that form during transcription (41). It is specifically recruited to R-loops that form near the 3′ ends of genes to facilitate efficient transcription termination, and its loss leads to read-through transcription (42, 43). Our work found that DHX9 resolves R-loops formed downstream of the poly(A) signal on HBV cccDNA transcripts, thereby facilitating transcription termination. DHX9 knockdown resulted in R-loop accumulation and RNA polymerase II retention at the HBV termination site. Beyond termination, we further establish that DHX9-mediated clearance of R-loops is a prerequisite for the subsequent nuclear export of the 3.5 kb HBV RNA, a step essential for viral core-associated DNA synthesis. This function of DHX9 in ensuring both efficient termination and cytoplasmic delivery of viral RNA reveals an integrated post-transcriptional checkpoint that HBV exploits to optimize its replication, highlighting DHX9 as a central host factor to support multiple stages of the HBV life cycle. Notably, whether DHX9 promotes the nucleocytoplasmic transport of other HBV RNA species remains to be investigated, which may explain its selective enhancement of HBV core-associated DNA levels.

In summary, our findings elucidate a novel mechanism by which the host helicase DHX9 promotes HBV replication through the resolution of R-loop structures during HBV cccDNA transcription. We provide evidence that HBV transcription induces R-loop accumulation, and that DHX9 resolves these DNA:RNA hybrids downstream of the poly(A) site, facilitating proper transcription termination of the 3.5 kb HBV RNA. This DHX9-mediated clearance of R-loops further promotes the nucleocytoplasmic transport of viral transcripts, ultimately enhancing the synthesis of core-associated DNA. These results highlight that targeting DHX9-mediated R-loops may contribute to antiviral strategies for chronic hepatitis B infection.

## MATERIALS AND METHODS

### Cell culture

Human hepatoma HepG2 cells were purchased from the American Type Culture Collection (ATCC). HepG2-NTCP cell line was a gift from Prof. Ningshao Xia (Xiamen University, China), and the HepAD38 cell line was kindly provided by Prof. C. Seeger (Institute for Cancer Research, Fox Chase Cancer Center, USA). PHHs were obtained commercially from Liver Biotech Company (Shenzhen, China). HepG2 and HepG2-NTCP cells were maintained in Dulbecco’s Modified Eagle Medium (DMEM) supplemented with 10% fetal bovine serum (FBS; NATOCOR, SFBE), 100 U/mL penicillin, and 100 μg/mL streptomycin (MedChemExpress, Cat. No. HY-K1006). For HepAD38 cells, the medium was used with the addition of 1 μg/mL tetracycline (Genview, AT341-10G) to suppress HBV transcription. PHHs were cultured in the specialized plating and maintenance media provided by the supplier. All cells were incubated at 37°C in a humidified atmosphere containing 5% CO_2_.

### Plasmids, specific small-interfering RNAs, and transfection

The HBV 1.1-mer plasmid, which contains a 1.1-fold overlength HBV genome (genotype D, accession no. V01460.1) under the control of the CMV promoter, was provided by Prof. Lin Lan (Army Medical University, China). The codon-optimized pcDNA3.1-DHX9-3HA and pcDNA3.1-RNase H1-3HA plasmids were constructed by Sangon Biotech (Shanghai, China). AAV-shDHX9 was generated by VectorBuilder (Guangzhou, China). The full-length cDNA of human DHX9 was cloned into the pSEB vector with a 3 × Flag tag located at the C-terminus. Specific small-interfering RNAs (siRNAs) targeting DHX9 were described previously and synthesized by Tsingke Biotech Company (Beijing, China) (44, 45). All primers and the target sequences of siRNAs are listed in Table S1. Transient transfections of plasmids and siRNAs were performed using Lipo8000 Transfection Reagent (Beyotime, C0533) following the manufacturer’s protocol.

### HBV particles production and cell infection

HBV particles were concentrated from HepAD38 cell supernatants by polyethylene glycol (PEG) precipitation (46). Briefly, culture supernatants were mixed with 5% PEG 8,000 and incubated with gentle rotation overnight at 4°C. The mixture was then centrifuged at 12,000 × *g* for 30 min at 4°C. The pellet was resuspended in serum-free Opti-MEM (Gibco, 31985-070) for subsequent infections.

For HepG2-NTCP cells and PHH infection, cells were inoculated with concentrated HBV particles at a multiplicity of infection (MOI) of 1,000 genome equivalents (GE) per cell. Infections were carried out in William’s E Medium (Gibco, Cat. No. 22551-022) supplemented with 2% DMSO, 1% glutamine, and 4% PEG 8,000. After 24 h, the cells were washed three times with phosphate-buffered saline (PBS; Dingguo, BF-0011).

### Enzyme-linked immunosorbent assay

HBsAg levels in mouse serum and cell culture supernatants were measured using enzyme-linked immunosorbent assay (ELISA) kits (Ke Hua Bio-engineering Company) according to the manufacturer’s protocols.

### Western blotting

Proteins were separated by SDS-polyacrylamide gel electrophoresis (SDS-PAGE) and transferred to polyvinylidene fluoride (PVDF) membranes (Millipore, Cat. No. IPVH00010). After blocking with 5% non-fat milk for 1 h at room temperature, membranes were incubated overnight at 4°C with the following primary antibodies: anti-Flag (Sigma-Aldrich, F3165), anti-HA (Invitrogen, 26183), anti-DHX9 (Santa Cruz, sc-137232), anti-β-actin (ZSGB-BIO, TA-09), anti-S9.6 (Mabnus, GS50001), and anti-HBc (ZSGB-BIO, ZM-0421). Following washes, membranes were incubated with appropriate horseradish peroxidase (HRP)-conjugated secondary antibodies. Proteins were detected using an enhanced chemiluminescence (ECL) substrate (Clarity Western ECL Substrate, Bio-Rad, 1705061). Band intensities were quantified using ImageJ software (National Institutes of Health, USA).

### RNA extraction and quantification

Total RNA was extracted from cultured cells or liver tissue using RNAiso Plus (TaKaRa, 9109) according to the manufacturer’s instructions. cDNA was synthesized with the PrimeScript RT Reagent Kit (TaKaRa, RR047A). Target transcripts were quantified by qPCR with 2× SYBR Green Master Mix (US Everbright, S2014L-A) and normalized to β-actin using the 2^−ΔΔCt^ method. Primer sequences are listed in Table S1.

### Extraction and quantification of HBV core-associated DNA

HBV core-associated DNA was isolated as previously reported (47). Briefly, cells were lysed in a buffer containing 10 mM Tris-HCl (pH 8.0), 1 mM EDTA, 1% NP-40, and 2% sucrose for 10 min at 37°C. After centrifugation at 15,000 × *g* for 5 min at room temperature, the cytoplasmic supernatant was collected and treated with micrococcal nuclease (New England Biolabs, M0247S). Core particles were precipitated with 5% PEG 8,000, and the pellet was digested with proteinase K (Sangon, A610451-0050). DNA was then purified by phenol/chloroform extraction followed by ethanol precipitation. Purified HBV core-associated DNA was quantified by qPCR using primers listed in Table S1.

For Southern blotting, HBV DNA was separated by electrophoresis, transferred to nylon membranes (Roche, 11417240001), and detected using the DIG Southern Starter Kit (Roche, 11585614910) following the supplier’s protocol.

### Extraction and quantification of HBV cccDNA

HBV cccDNA was isolated using a modified Hirt extraction procedure as previously described (47). Briefly, cells were lysed in 50 mM Tris-HCl (pH 8.0), 10 mM EDTA, 1% SDS, and 150 mM NaCl. The lysate was mixed with 2.5 M KCl and incubated overnight at 4°C with gentle rotation. DNA was then purified by sequential phenol-chloroform extraction and ethanol precipitation. To remove residual linear and single-stranded DNA contaminants, the extracted DNA was treated with Plasmid-safe ATP-Dependent DNase (Epicentre, E3101K). Absolute quantification of HBV cccDNA was performed by qPCR using primers listed in Table S1.

### Immunofluorescence

Cells grown on glass coverslips were fixed with 4% paraformaldehyde, permeabilized with 0.5% Triton X-100, and blocked with 5% goat serum. After incubation with primary antibodies overnight at 4°C, samples were probed with Alexa Fluor 488- or 546-conjugated secondary antibodies for 2 h at room temperature. Nuclei were counterstained with 4′,6-diamidino-2-phenylindole (DAPI). Images were acquired using a Leica TCS SP8 confocal laser scanning microscope.

### Dot blot

Cells were harvested at 48 h post-transfection. The pellets were collected by centrifugation at 5,000 × *g*, 4°C for 10 min, and lysed in 500 μL DNA lysis buffer (0.1 M NaCl, 0.05 M Tris-HCl [pH 8.0], 1% SDS, 0.025 M EDTA) with 5.5 μL proteinase K (20 mg/mL) at 37°C for 16 h. Nucleic acids were precipitated by adding 214 μL 5 M NaCl and centrifuged at 12,000 × *g* for 30 min, 4°C. DNA in the supernatant was precipitated with ice-cold isopropanol, washed with 70% ethanol, and dissolved in 20 μL TE buffer (10 mM EDTA, 50 mM Tris/HCl). 4 μg DNA:RNA hybrid was incubated with 5 U RNase H (Beyotime, Cat. No. R7088S) in 0.5 μL 10× buffer (total volume 5 μL) at 37°C for 20 min, followed by termination with 1 μL 0.5 M EDTA. For dot blot, samples were spotted onto a nitrocellulose membrane, UV-crosslinked, and blocked with 5% BSA. The membrane was incubated with S9.6 antibody at 4°C overnight. After incubation with HRP-conjugated goat anti-mouse antibody for 1 h at room temperature, the signals were detected using an enhanced ECL substrate.

### DRIP

Cells were harvested from 10 cm dishes by scraping in cold 1× PBS, followed by centrifugation at 700 × *g* for 2 min. The pellet was lysed in 500 μL DNA lysis buffer, incubated with 5 μL proteinase K (20 mg/mL) at 37°C for 16 h, and extracted twice with phenol:chloroform:isoamyl alcohol (25:24:1). DNA was precipitated with 2.5 volumes of ice-cold ethanol, washed with 75% ethanol, and dissolved in 50 μL TE buffer. For DRIP, 50 μg DNA was sheared by sonication to 200–1,000 bp fragments, purified, and divided into RNase H^+^ and RNase H^–^ groups. The RNase H^+^ sample was treated with 10 U RNase H at 37°C for 1 h. The two groups were immunoprecipitated using protein A/G beads pre-coated with S9.6 antibody (or IgG control). DNA:RNA hybrids were analyzed by qPCR. For DRIP-seq, HepAD38 (Tet^−^) cells were transfected with the indicated plasmids. After 48 h, the cells were sent to Aksomics Inc. (Shanghai, China). The resulting sequencing reads were aligned to the reference genome (48).

### ChIP

Cells were fixed with formaldehyde at a final concentration of 1% for 10 min at room temperature, followed by quenching with 500 μL of 2.5 M glycine for 5 min. After washes with PBS containing protease inhibitors (C0001, TargetMol), the cells were scraped in 500 μL of PBS and pelleted by centrifugation at 700 × *g* for 5 min at 4°C. The cell pellet was resuspended in 500 μL ChIP Lysis Buffer (50 mM HEPES, 150 mM NaCl, 1 mM EDTA, 0.1% SDS, 0.1% sodium deoxycholate, 1% Triton X-100, pH 7.5) with 1% protease inhibitors and lysed on ice for 30 min. Chromatin was sheared by sonication to generate 200–1,000 bp fragments, and the lysate was clarified by centrifugation at 13,000 × *g*, 4°C for 15 min.

For immunoprecipitation, protein A/G beads were incubated with the clarified lysate for 1 h at 4°C. The sample was divided into three aliquots: one incubated with 5 μg antibody, one with IgG control overnight at 4°C, and the third reserved as Input. Fresh beads were added to the antibody-treated samples and rotated for 4 h at 4°C. The beads were washed with high-salt buffer (50 mM HEPES, 500 mM NaCl, 1 mM EDTA, 1% SDS, 1% Triton X-100, 0.1% sodium deoxycholate, pH 7.5). The complexes were eluted twice with pre-warmed ChIP elution buffer (10 mM EDTA, 50 mM Tris-HCl, 2% SDS, pH 7.5). Eluted DNA was reverse-cross-linked with 5 M NaCl at 65°C for 4 h, purified by phenol-chloroform extraction and ethanol precipitation, and analyzed by qPCR.

### Nuclear and cytoplasmic RNA fractionation

Cells were resuspended in hypotonic buffer (25 mM Tris-HCl, 1 mM MgCl_2_, 5 mM KCl, pH 7.4) and kept on ice for 5 min. An equal volume of the same buffer supplemented with 1% NP-40 was then added, followed by another incubation on ice for 5 min. Following centrifugation at 5,000 × *g* for 5 min, the supernatant was retained as the cytosolic fraction. The resulting pellet was washed twice with hypotonic buffer and subsequently resuspended in nuclear resuspension buffer (20 mM HEPES, 400 mM NaCl, 1 mM EDTA, 1 mM EGTA, 1 mM DTT, 1 mM PMSF, pH 7.9). After an incubation on ice for 30 min, the samples were centrifuged at 12,000 × *g* for 10 min to collect the supernatant and designated as the nuclear fraction. RNAs were reverse-transcribed and detected by qPCR. The primers employed in this assay are provided in Table S1.

### Animal experiments

*Alb-Cre* Tg C57BL/6 mice expressing Cre recombinase under the albumin promoter were purchased from Gempharmatech Company (Jiangsu, China). The mice (6 weeks old, five individuals per group) were randomly injected with AAV recombinant adenovirus AAV-shControl and AAV-shDHX9 via tail veins, respectively. After 3 weeks, mice were hydrodynamically injected with 4 μg prcccDNA plasmid diluted to 2 mL of PBS via tail veins. One week later, serum HBsAg was determined by ELISA to confirm successful HBV infection in mice. One week after the injection, liver tissues and sera were collected for further analysis. All animals were raised by the Laboratory Animal Center of Chongqing Medical University and approved by the Animal Ethics Committee of Chongqing Medical University (IACUC-CQMU-2023-10005).

### Statistical analysis

All data were analyzed using GraphPad Prism software (version 8.3.0). Quantitative results are presented as the mean ± standard deviation from three independent experimental replicates. Normality was assessed using the Shapiro-Wilk test, and homogeneity of variances was verified by the Brown-Forsythe test. Comparisons between two groups were performed using an unpaired two-tailed Student’s *t*-test. For comparisons among multiple groups, one-way ANOVA was applied, followed by Tukey’s *post hoc* test. *P* < 0.05 was considered statistically significant.

## Data Availability

The DRIP-seq data has been deposited in the OMIX database, China National Center for Bioinformation/Beijing Institute of Genomics, Chinese Academy of Sciences (https://ngdc.cncb.ac.cn/omix: accession number OMIX020480).

## SUPPLEMENTAL MATERIAL

Supplemental material mbio.01449-26-s0001.docx Fig. S1 to S3; Table S1.

ASM does not own the copyrights to Supplemental Material that may be linked to, or accessed through, an article. The authors have granted ASM a non-exclusive, world-wide license to publish the Supplemental Material files. Please contact the corresponding author directly for reuse.
