# Supplementary material for: Resolution of viral R-loop by DHX9 promotes hepatitis B virus replication
Source: mBio. 2026 Sep 18;17(10):e01449-26. doi: 10.1128/mbio.01449-26 (PMC13712953; doi:10.1128/mbio.01449-26)
Supplement: Supplementary file 1 [file mbio.01449-26-s0001.docx]

**Supplemental Figures and Tables**

**Resolution of viral R-loop by DHX9 promotes hepatitis B virus replication**

Yubo Pi^1^, Huimin Luo^1^^,2^, Kai Wang^1^, Ni Tang^1^, Ailong Huang^1,*^, Yanmeng Chen^3,*^

^1^Key Laboratory of Molecular Biology for Infectious Diseases, Ministry of Education, Department of Infectious Diseases, Institute for Viral Hepatitis, The Second Affiliated Hospital of Chongqing Medical University, Chongqing, China.

^2^Youyang Hospital, A Branch of The First Affiliated Hospital of Chongqing Medical University.

^3^Key Laboratory of Laboratory Medical Diagnostics, Ministry of Education, Department of Laboratory Medicine, Chongqing Medical University, Chongqing, China.

^*^Address correspondence to Yanmeng Chen, [yanmengchen@cqmu.edu.cn](mailto:yanmengchen@cqmu.edu.cn); Ailong Huang, [ahuang@cqmu.edu.cn](mailto:ahuang@cqmu.edu.cn)

Yubo Pi and Huimin Luo contributed equally to this article.

**Supplemental Figure 1. Overexpression of DHX9 enhances HBV replication in HepG2-NTCP.**

HepG2-NTCP cells were infected with HBV particles. After 24h post infection, the cells were transfected with increasing amounts of HA-tagged DHX9 plasmids. (A) Western blot analysis of HA-DHX9 expression in HepG2-NTCP cells. (B) The levels of HBV core-associated DNA in HepG2-NTCP cells were determined by Southern blot. (C) HBsAg secretion measured by ELISA in culture supernatants of HepG2-NTCP cells. (D) RT-qPCR analysis of 3.5 kb HBV RNA levels in HepG2-NTCP cells. (E) Quantification of HBV cccDNA levels in HepG2-NTCP cells using qPCR. (F) The ratio of 3.5 kb HBV RNA to HBV cccDNA was calculated to assess HBV cccDNA transcriptional activity. Data represent ± SD from three biological replicates. ns: no significance, one-way ANOVA with Tukey's post hoc test.

**Supplemental Figure 2. Knockdown of DHX9 inhibits HBV replication in HepG2-NTCP.**

HepG2-NTCP cells were infected with HBV particles. After 24 h post-infection, the cells were transfected with siDHX9 or siControl as indicated. (A) Western blot analysis of DHX9 expression was used to confirm the knockdown efficiency. (B) The levels of HBV core-associated DNA in HepG2-NTCP cells were determined by Southern blot. (C) The levels of HBsAg in culture medium were determined by ELISA. (D) Quantification of 3.5 kb HBV RNA levels using RT-qPCR. (E) Quantification of HBV cccDNA levels in HepG2-NTCP cells using qPCR. (F) The ratio of 3.5 kb HBV RNA to HBV cccDNA was calculated to assess HBV cccDNA transcriptional activity. Data represent ± SD from three biological replicates. ns: no significance. Two-tailed unpaired Student's t test.

**Supplemental Figure 3. DRIP-seq reveals R-loop formation during HBV transcription.**

(A) The schematic depiction of DRIP-seq. (B) DRIP-seq was used to detect R-loop formation of HBV transcripts in HepAD38 cells.

**Supplemental Table 1. Sequence of Oligonucleotides.**

| **Name** | **Sequence (5’-3’)** |
| --- | --- |
| **Primer sequences for qPCR** | |
| HBV cccDNA | Forward: CTCCCCGTCTGTGCCTTCT |
|  | Reverse: GCCCCAAAGCCACCCAAG |
| HBV core-associated DNA (F4) | Forward: TGCGGCGTTTTATCATATTCC |
|  | Reverse: ATACCTTGGTAGTCCAGAAGAACCA |
| 3.5 kb HBV RNA (F2) | Forward: CCTAGTAGTCAGTTATGTCAAC |
|  | Reverse: TCTATAAGCTGGAGGAGTGCGA |
| γ-actin | Forward: TTCGTGGGCTGGTGAGAAAA |
|  | Reverse: TTGGGAGTGCCTTGTA |
| HBV F1 | Forward: ATTTGGAGCTACTGTGG |
|  | Reverse: TTGCCTGAGTGCAGTATGG |
| HBV F3 | Forward: CATTTACACCAAGACATTA |
|  | Reverse: ATGCCCGCCTTCCATAG |
| HBV F5 | Forward: ATGGCTGCTARGCTGTGCTGCCAA |
|  | Reverse: AAGTGCACACGGTYYGGCAGAT |
| **Primer sequences for molecular cloning** | |
| pSEB-Flag-DHX9 | Forward:  ATTTGCGGCCGCACCATGGGAGATGTGAAAAATTTTC |
|  | Reverse: CGCGGATCCGTAGCCGCCGCCGCCGCGGCCC |
| **Sequences for siRNA** | |
| siDHX9#1 | Sense: AAGAAGUGCAAGCGACUCUAG |
|  | Anti-Sense: CUAGAGUCGCUUGCACUUCUU |
| siDHX9#2 | Sense: CGACAAUGGAAGCGGAUAU |
|  | Anti-Sense: AUAUCCGCUUCCAUUGUCG |
| siControl | Sense: UUCUCCGAACGUGUCACGU |
|  | Anti-Sense: ACGUGACACGUUCGGAGAA |
